# Environmental and occupational respiratory diseases -1035. Isolation and purification of major allergenic protein of moth and rice weevil insect extracts

**DOI:** 10.1186/1939-4551-6-S1-P34

**Published:** 2013-04-23

**Authors:** Mohd Adnan Kausar, Waseem Ahmad Siddiqui

**Affiliations:** 1 Department of Biochemistry, Jamia Hamdard, New Delhi, India

## Background

The study was plan to isolate and purify the major allergenic protein from moth and rice weevil insect whole body extracts (WBEs).

## Methods

For purification of allergenic proteins, crude insect WBEs were subjected to 80% ammonium sulphate precipitation, anion exchange column chromatography using DEAE-Sephacel and fast protein liquid chromatography (FPLC) using Mono Q column.

## Results

After 80% ammonium sulphate precipitation, most of the proteins were recovered in 80% precitable fraction, 58.4% in moth and 71.2% in rice weevil. However, the recovery in supernatant was low 6.1% in rice weevil and 14.4 in moth extract. In ELISA inhibition of both the insect WBEs, most of the allergenic activity was recovered in 80% ammonium sulphate precipitate fraction (80% ppt). No allergenic protein bands were detected in the supernatants of both insect extracts. Molecular weights of allergenic proteins ranged from 10 kDa in to 105 kDa.

When 80%ppt of both the insect extracts was subjected to anion exchange chromatography, three peaks were obtained in each extracts. FrI of both the purified insects induced significant inhibition of insects ELISA. FrII of both insects also produced some inhibition, FrIII failed to induce any inhibition. SDS-PAGE analysis of FrI revealed that it contains 13 proteins in moth and 10 protein bands in rice weevil. In immunoblot experiments, FrI showed only one allergenic protein of moth (30kDa) and three in rice weevil (63kDa, 45kDa and 22kDa).

On FPLC of FrI of moth and rice weevil extract, four fractions (FrIa, FrIb, FrIc and FrId) were obtained. Of the 4 fractions, FrIa of moth and FrIc of rice weevil showed maximum allergenic activity.In SDS-PAGE analysis of FrIa of moth and FrIc of rice weevil revealed protein bands of 30kDa and 45 kDa, respectively. Major allergenic proteins of moth extract identified in immunoblot experiments, corresponding to molecular weight 30 kd was recovered in fraction moth-FrIa; in rice weevil extract major allergenic protein was recovered in fractions FrIc of molecular weight 45kDa.

## Conclusions

This purified protein may be used as a reference reagent for the standardization of insect extracts used for diagnosis and immunotherapy of allergic patients.

